# A Bioactive Olive Pomace Extract Prevents the Death of Murine Cortical Neurons Triggered by NMDAR Over-Activation

**DOI:** 10.3390/molecules25194385

**Published:** 2020-09-24

**Authors:** Alice Franchi, Marco Pedrazzi, Alessandro Alberto Casazza, Enrico Millo, Gianluca Damonte, Annalisa Salis, Nara Liessi, Franco Onofri, Antonella Marte, Silvia Casagrande, Roberta De Tullio, Patrizia Perego, Monica Averna

**Affiliations:** 1 Department of Experimental Medicine (DIMES), University of Genoa, Viale Benedetto XV-1, 16132 Genova, Italy; alice.franchi90@gmail.com (A.F.); marco.pedrazzi@unige.it (M.P.); enrico.millo@unige.it (E.M.); gianluca.damonte@unige.it (G.D.); franco.onofri@unige.it (F.O.); antonella.marte@unige.it (A.M.); silvia.casagrande@unige.it (S.C.); detullio@unige.it (R.D.T.); 2 Department of Civil, Chemical and Environmental Engineering, University of Genoa, Pole of Chemical Engineering, via Opera Pia 15, 16145 Genoa, Italy; alessandro.casazza@unige.it (A.A.C.); p.perego@unige.it (P.P.); 3 Centre of Excellence for Biomedical Research (CEBR), University of Genoa, Viale Benedetto XV 9, 16132 Genova, Italy; annalisa.salis@unige.it (A.S.); liessi_nara@libero.it (N.L.); 4 IRCCS Ospedale Policlinico San Martino, 16132 Genova, Italy

**Keywords:** calcium homeostasis, NMDAR, neuron primary culture, calpain, olive pomace extract, proanthocyanidins

## Abstract

We have recently demonstrated that bioactive molecules, extracted by high pressure and temperature from olive pomace, counteract calcium-induced cell damage to different cell lines. Here, our aim was to study the effect of the same extract on murine cortical neurons, since the preservation of the intracellular Ca^2+^-homeostasis is essential for neuronal function and survival. Accordingly, we treated neurons with different stimuli in order to evoke cytotoxic glutamatergic activation. In these conditions, the high-pressure and temperature extract from olive pomace (HPTOPE) only abolished the effects of *N*-methyl-d-aspartate (NMDA). Particularly, we observed that HPTOPE was able to promote the neuron rescue from NMDA-induced cell death. Moreover, we demonstrated that HPTOPE is endowed with the ability to maintain the intracellular Ca^2+^-homeostasis following NMDA receptor overactivation, protecting neurons from Ca^2+^-induced adverse effects, including aberrant calpain proteolytic activity. Moreover, we highlight the importance of the extraction conditions used that, without producing toxic molecules, allow us to obtain protecting molecules belonging to proanthocyanidin derivatives like procyanidin B2. In conclusion, we can hypothesize that HPTOPE, due to its functional and nontoxic properties on neuronal primary culture, can be utilized for future therapeutic interventions for neurodegeneration.

## 1. Introduction

Growing evidence [1,2,3,4,5] has shown that a dietary pattern inspired by the Mediterranean diet (MD) principles is associated with numerous health benefits, such as protecting against neurodegeneration. The beneficial effects of MD are probably due to the abundant consumption of extra virgin olive oil (EVOO), known to be rich in phenolic compounds and endowed with different nutritional properties and health effects [6]. The phenolic compounds include a wide variety of structures expanding from the simplest structures, such as phenolic acids and alcohols, to the most complex oligomeric ones, such as proanthocyanidins. All these compounds are also present in olive oil production wastes, such as olive pomace and olive mill waste waters [7]. Many studies have demonstrated that polyphenols are endowed with a lot of different biological properties, such as anti-inflammatory and antioxidant activity or cardioprotective and neuroprotective activity [8,9,10,11,12]. However, although numerous studies have shown that the polyphenols can protect neuronal cells against excitotoxicity, the exact mechanism remains unclear. Particularly, studies have shown that some of these compounds protect neurons by reducing glutamate-mediated Ca^2+^ influx inhibiting Protein Kinase C activity and the subsequent phosphorylation of NR1 of the *N*-methyl-d-aspartate (NMDA) receptor (NMDAR) [13]. Other studies [14,15] reported an inhibitory effect of some polyphenols on cytotoxic NO production induced by cell exposure to pathological concentrations of quinolinic acid, an activator of the NMDAR, providing evidences for the beneficial effects of polyphenols in excitable tissues, such as the central nervous system. It is well-known that chronic neurodegenerative diseases, such as Alzheimer’s and Parkinson’s disease, share common pathological characteristics, including the overactivation of NMDAR, with a consequent alteration of the intracellular Ca^2+^ homeostasis, as the primary cause of neuronal death [16,17,18,19]. Moreover, when the calcium homeostasis is compromised, the persistent activation of the proteolytic calcium-dependent protease calpain, known to be involved in the development of neurodegeneration, leads to the uncontrolled degradation of its substrates and, finally, to cell death [20]. For this reason, the inhibition of calpain is considered an interesting target to prevent neurodegenerative disease associated with neuronal loss [21,22,23,24].

We have recently observed that bioactive molecules, extracted from olive pomace of the Taggiasca cultivar by means of a high-pressure and temperature (HPT) reactor whose operation is based on a green extraction technique [7,25], counteracted calcium-induced cell damage to different cell lines [26], inhibiting also the aberrant calpain activity. Accordingly with these results, and since the preservation of the intracellular Ca^2+^ homeostasis is essential for neuronal survival, the aim of our study is to investigate if the high-pressure and temperature extract from olive pomace (HPTOPE) could counteract the glutamate-induced murine cortical neuron death by regulating the intracellular dynamics of calcium. In this regard, we demonstrated that HPTOPE is able to maintain intracellular calcium homeostasis following the overactivation of NMDAR. This effect is fundamental for protecting neurons from calcium-induced adverse effects, including the pathological activation of calpain, involved in the phenomena of neurodegeneration.

## 2. Results

### 2.1. Effect of HPTOPE on Neuronal Death Induced by Ionotropic Glutamate Receptor Agonists

**Figure 1:**
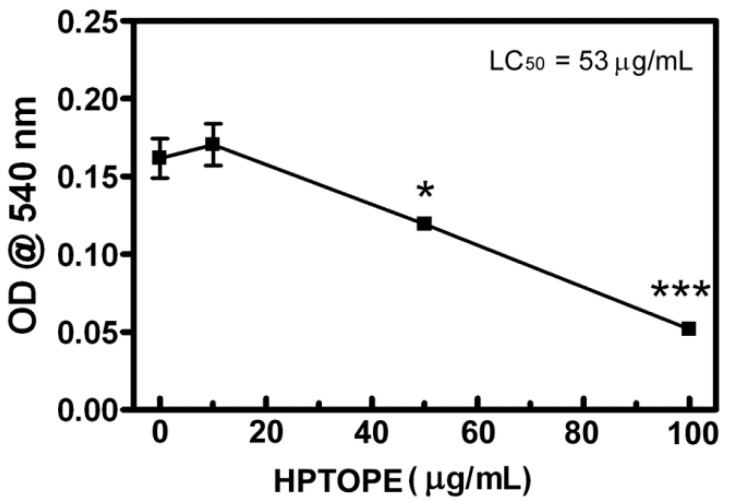
Cell viability of cortical neurons exposed to increasing concentrations of high-pressure and temperature extract from olive pomace (HPTOPE). After 14 days in vitro (DIV), neurons were incubated for 24 h with the indicated HPTOPE concentrations. Cell viability was evaluated by neutral red uptake (NRU) assay. Values represent means ± SEM from three independent experiments in triplicate. * *p* < 0.05 and *** *p* < 0.001 vs. control, according to ANOVA, followed by Tukey’s post-hoc test. LC_50_ was calculated using the online free tool “Quest Graph™ LC_50_ Calculator”, AAT Bioquest, Inc., 9 Sep. 2020, https://www.aatbio.com/tools/lc50-calculator.

**Figure 2:**
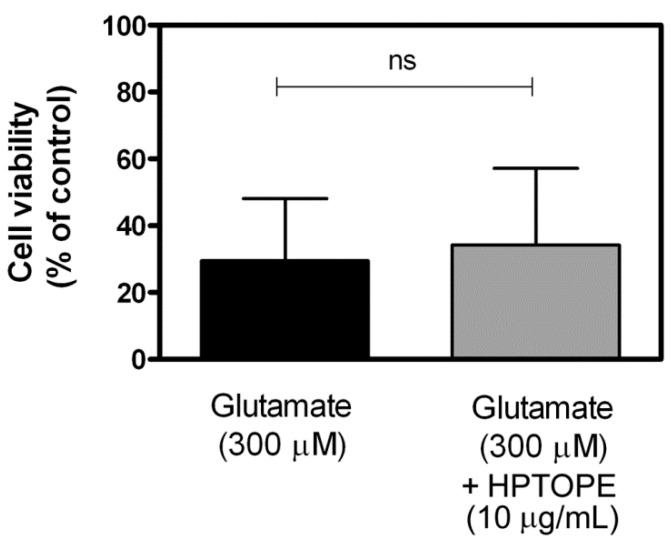
Cell viability of cortical neurons exposed to glutamate in the presence of HPTOPE. After 14 DIV, neurons were treated for 24 h with the indicated stimuli. Cell viability was evaluated by NRU assay. Values represent means ± SEM from nine independent experiments in triplicate. ns, not statistically significant, according to *t*-test.

**Figure 3:**
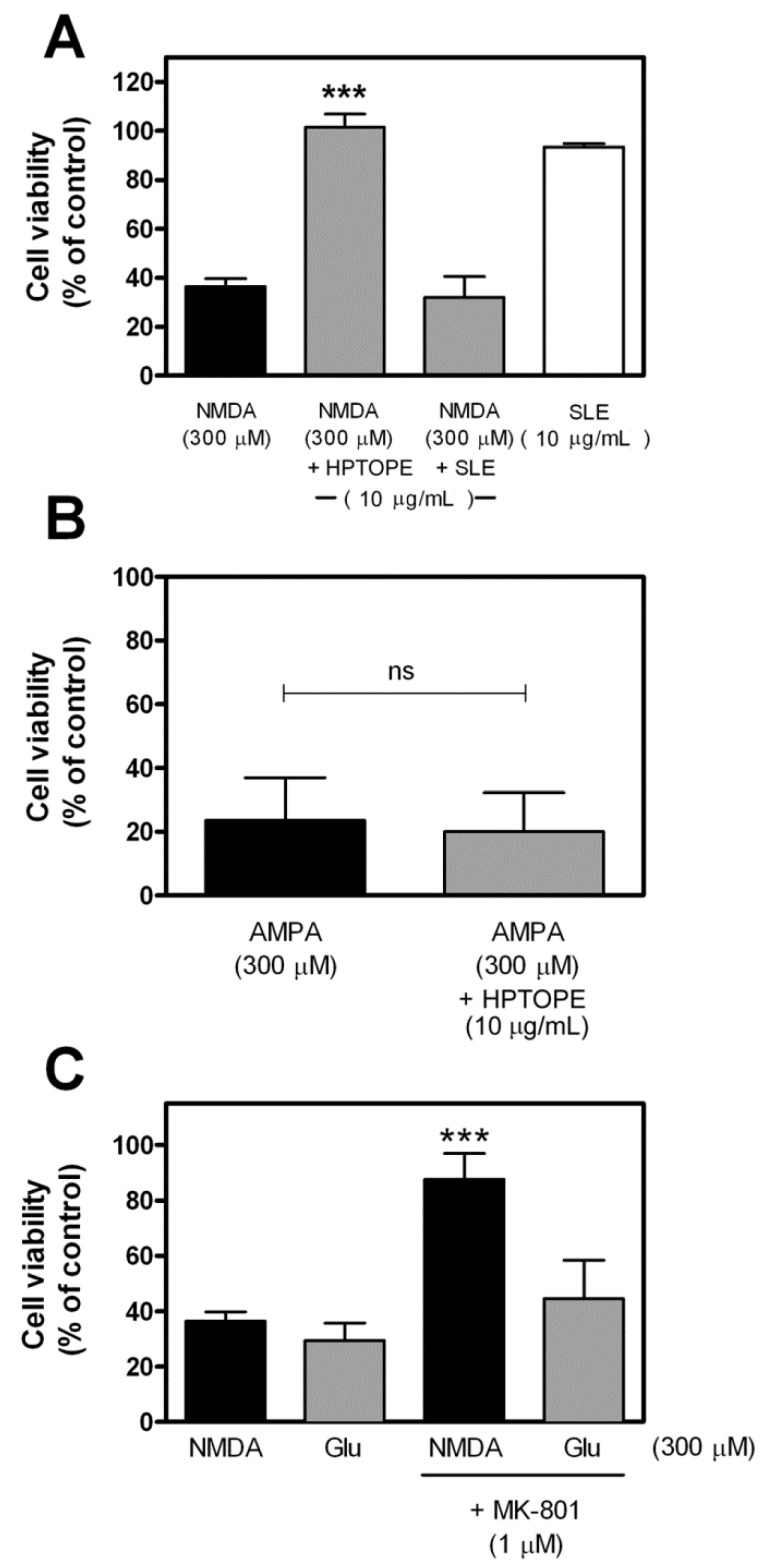
Cell viability of cortical neurons following glutamate receptor stimulation in the presence of either HPTOPE or a solid–liquid extract (SLE). After 14 DIV, neurons were treated for 24 h at 37 °C with the indicated stimuli. Cell viability was evaluated by NRU assay. (**A**) Data are means ± SEM from fourteen (*N*-methyl-d-aspartate (NMDA) and NMDA + HPTOPE) or three (NMDA + SLE and SLE) independent experiments in triplicate. *** *p* < 0.001, according to ANOVA, followed by Tukey’s post-hoc test. (**B**) Values represent means ± SEM from two independent experiments in duplicate. ns, not statistically significant, according to *t*-test. AMPA: α-amino-3-hydroxy-5-methyl-4-isoxazolepropionic acid. (**C**) Data are means ± SEM from fourteen (NMDA), nine (Glu), six (Glu + dizocilpine (MK–801)), or five (NMDA + MK-801) independent experiments in triplicate. *** *p* < 0.001 vs. NMDA, according to ANOVA, followed by Tukey’s post-hoc test.

After 14 days in vitro (DIV), murine primary cortical neurons were first exposed to increasing concentrations of HPTOPE to establish the maximum noncytotoxic concentration to be used in all our experiments. As shown in Figure 1, we observed that, after 24 h of incubation, the maximum noncytotoxic concentration of HPTOPE, determined by a neutral red uptake (NRU) assay, was 10 µg/mL. Since the cytotoxic effects of increasing the Ca^2+^ influx in neurons are known [27,28,29] to be an important cause of neuronal death, we treated the cells with 300-µM glutamate for 24 h [30,31], in order to induce a toxic influx of Ca^2+^ in the cortical neurons. As reported in Figure 2, differently from that previously observed in cell lines [26], HPTOPE was not able to protect neurons from glutamate-induced cell death. This result suggests that HPTOPE, being not able to protect neurons from death caused by nonspecific glutamate stimulation, could probably act through a mechanism that involves other or more precise molecular targets. For this purpose, we stimulated neurons with the agonists of the two major expressed ionotropic glutamate receptors in these cells: NMDA and α-amino-3-hydroxy-5-methyl-4-isoxazolepropionic acid (AMPA) receptors [32], both able to promote cellular Ca^2+^ influx. First, we treated neurons with cell toxic amounts of NMDA for 24 h, in the absence or in the presence of 10 µg/mL of HPTOPE. As shown in Figure 3A, when neurons were selectively stimulated with 300-µM NMDA, HPTOPE significantly prevented cell death. For comparison, the lack of a protective effect of the solid–liquid extract (SLE), a classic olive pomace extract obtained at room temperature, as indicated in the Materials and Methods section, was also reported in Figure 3A, highlighting the importance of the extraction method from olive pomace to obtain the bioactive molecule(s) endowed with protective neuronal properties. Conversely, as shown in Figure 3B, when we stimulated neurons using AMPA instead of NMDA, HPTOPE was no longer able to prevent cell death, as previously observed under glutamate cell stimulation conditions. These results suggest that the bioactive molecule(s), contained only in HPTOPE, exert their protective effect through a selective interaction with the NMDAR signaling pathway. To support this conclusion, we treated again the neurons with toxic amounts of both NMDA or glutamate for 24 h in the presence or absence of dizocilpine (MK-801), a selective antagonist of NMDAR [33,34]. As shown in Figure 3C, MK-801, just like HPTOPE, was unable to counteract cell death caused by glutamate but significantly protected neurons from death caused by stimulation with NMDA. 

### 2.2. Effect of HPTOPE on Intracellular Calcium Homeostasis in Conditions of NMDAR Overactivation

**Figure 4:**
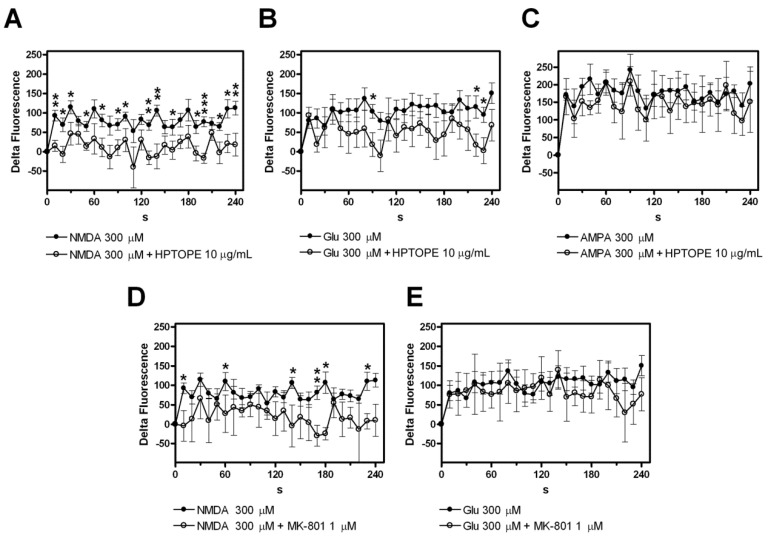
Evaluation of [Ca^2+^]_i_ increase in cortical neurons exposed to cytotoxic stimuli in the presence of HPTOPE. Calcium Green™-1AM(CG)-loaded neurons were treated with the indicated agonists in the absence (●) or in the presence (*O*) of 10-µg/mL HPTOPE or 1-µM MK-801 for the indicated time at 37 °C. CG-dependent fluorescence was monitored every 10 s. [Ca^2+^]_i_ increase is expressed as “delta fluorescence”, which is the difference between the CG-dependent fluorescence of the stimulated samples and the ones of the vehicle-treated samples, both measured at each recording time and subtracted by the one measured at the starting time. (**A**) Data are means ± SEM from eleven (●) or six (*O*) independent experiments in triplicate. (**B**) Data are means ± SEM from ten (●) or six (*O*) independent experiments in triplicate. (**C**) Data are means ± SEM from four independent experiments in triplicate. (**D**) Data are means ± SEM from eleven (●) or three (*O*) independent experiments in triplicate. (**E**) Data are means ± SEM from ten (●) or four (*O*) independent experiments in triplicate. * *p* < 0.05, ** *p* < 0.01, and *** *p* < 0.001 according to *t*-test.

To better characterize the events that underlie the protective effect of HPTOPE on neurons, under conditions of aberrant NMDAR stimulation, we measured the intracellular Ca^2+^ concentration changes. As shown in Figure 4, although the stimulation of all glutamatergic receptors analyzed resulted in an increase in intracellular [Ca^2+^], HPTOPE was able to significantly reduce this increase only during the selective NMDAR stimulation (Figure 4A) but not under glutamate and AMPA stimulation conditions (Figure 4B,C). Results similar to those reported in Figure 4A were obtained when the cells were stimulated with NMDA in the presence of MK-801 (Figure 4D). All these results confirm that HPTOPE preserves the neuronal viability acting specifically on the calcium influx through NMDAR. Furthermore, MK-801 failed to block the intracellular Ca^2+^ influx promoted by glutamate stimulation (Figure 4E), demonstrating that, under these conditions, NMDAR is not the only receptor responsible for the glutamate-mediated Ca^2+^ influx leading to neuronal death (see Figure 2).

**Figure 5:**
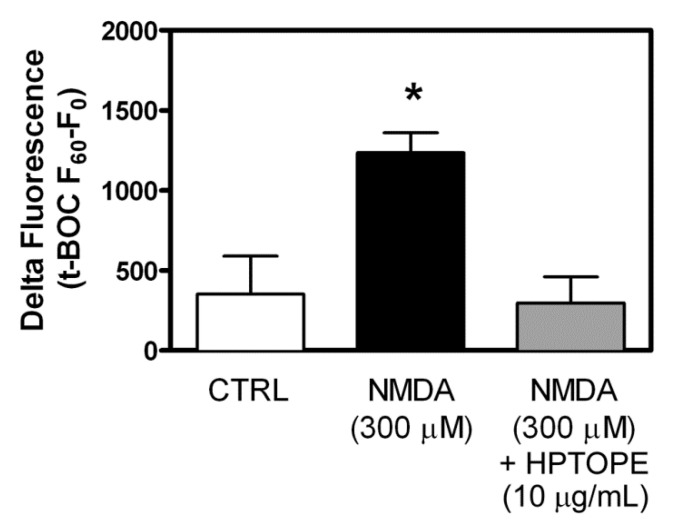
Evaluation of calpain-1 activity in cortical neurons following NMDA receptor (NMDAR) stimulation in the presence of HPTOPE. *t*-butyloxycarbonyl (*t*-BOC)-loaded neurons were treated with the indicated stimuli. The protease activity is expressed as “delta fluorescence”, which is calculated as the difference between the *t*-BOC-dependent fluorescence measured at 60 min (F_60_) and the one recorded at the starting time (F_0_). Data are means ± SEM from five independent experiments in triplicate. * *p* < 0.05 vs. control and ** *p* < 0.01 vs. NMDA, according to ANOVA, followed by Tukey’s post-hoc test.

Since HPTOPE was able to counteract the neuronal death promoted by intracellular calcium overloading, and since it has been reported that the aberrant proteolytic activity of the calcium-dependent protease calpain is involved in calcium-induced neuronal death [35,36,37,38], we investigated if HPTOPE could also affect the activity of this protease. As shown in Figure 5, in conditions of toxic NMDAR stimulation, the increase in intracellular calcium concentration, due to the receptor opening, resulted in a significant rise in the protease activity. Conversely, when we treated neurons with NMDA in the presence of HPTOPE, the inhibition of the calcium influx exerted by the extract resulted in the significant prevention of the aberrant activation of calpain. These results provided further evidence that HPTOPE is endowed with the ability to maintain intracellular calcium homeostasis, protecting neurons from different calcium-induced adverse effects, including aberrant calpain activation.

**Figure 6:**
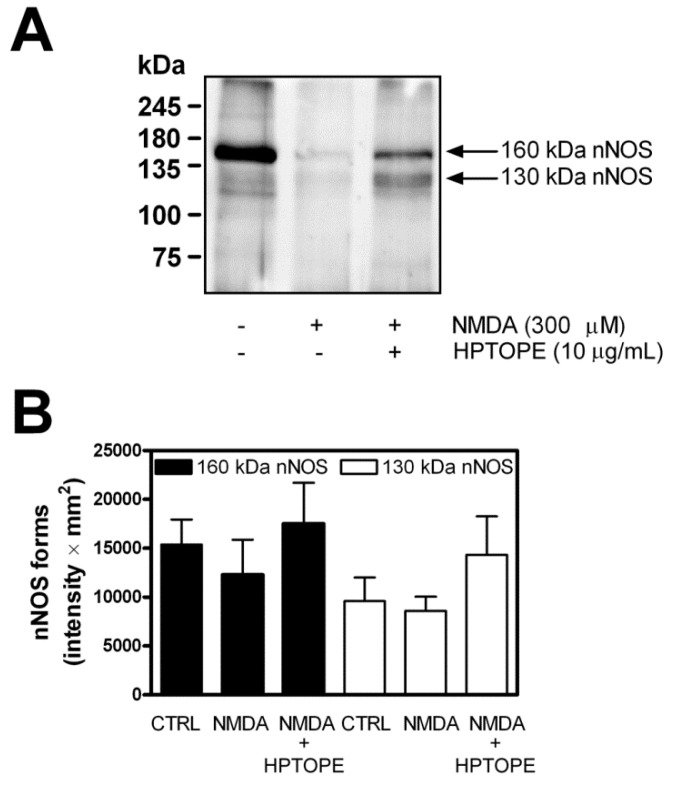
Evaluation of neuronal nitric oxide synthase (nNOS) in cortical neurons following NMDAR stimulation in the presence of HPTOPE. After 14 DIV, neurons were incubated for 4 h with the indicated stimuli. Aliquots of total proteins from neuron lysates were submitted to 8% SDS/PAGE followed by immunoblotting for nNOS. (**A**) A representative blot of nine is shown. (**B**) Relevant immunoreactive bands were quantified, normalized vs. β-actin signal, and reported as intensity × mm^2^. Data are means ± SEM from nine independent experiments.

To further confirm that HPTOPE prevented the aberrant proteolytic activation of calpain, we analyzed the peculiar calpain-mediated digestion pattern of neuronal nitric oxide synthase (nNOS). In this regard, we have previously demonstrated that the conversion of the native 160-kDa form of nNOS into the activated form of 130 kDa is a calcium-dependent event mediated by the physiological proteolytic activity of calpain that allows the modulation of NO production [39,40]. Accordingly, as reported in Figure 6, in neurons treated with toxic amounts of NMDA, we observed a trend for a decrease of both the native and activated nNOS forms. Conversely, in neurons treated with NMDA in the presence of HPTOPE, we observed, further than the preservation of the native form of nNOS, a trend for an accumulation of the activated 130 kDa nNOS form, confirming that HPTOPE prevents the aberrant proteolytic activity of the calcium-dependent protease calpain, allowing it to maintain its physiological function.

Taken together, all these results definitely indicate that HPTOPE is able to protect neurons from those intracellular calcium-induced harmful events promoted by the overactivation of NMDAR, including the aberrant calpain activation.

### 2.3. Identification of the Possible Bioactive Molecule(s) Contained in HPTOPE

**Figure 7:**
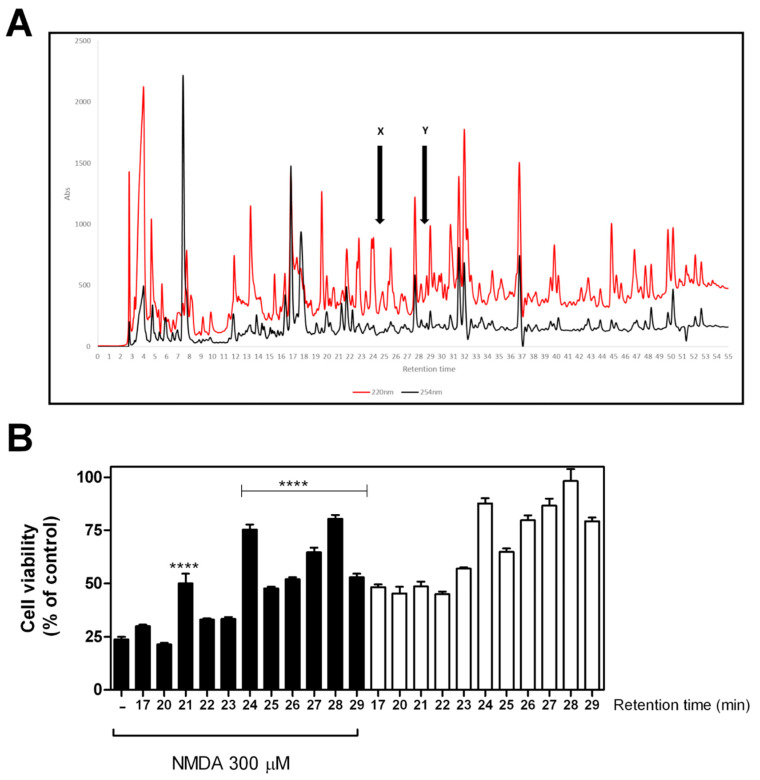
Assay of the chromatographic fractions of HPTOPE. After 14 DIV, neurons were incubated for 24 h in the presence of the different chromatographic fractions of HPTOPE (corresponding to the indicated retention time). (**A**) HPTOPE (20 µL) was fractionated by means of HPLC, as specified in Methods, and elution was monitored at 220 (red) and 254 (black) nm. The X and Y arrows indicate the compounds discussed in the text. (**B**) An aliquot (1/10) of each reconstituted 100-µL fraction was assayed in the presence (black bars) or in the absence (white bars) of NMDA. Cell viability was evaluated by NRU assay. Values represent means ± SEM from one assay in triplicate. **** *p* < 0.0001 vs. NMDA (first black bar), according to ANOVA, followed by Tukey’s post-hoc test.

Thus, to identify the molecular nature and structure of the bioactive molecule(s) contained in HPTOPE, we fractionated HPTOPE and tested the biological protective activity of the fractions corresponding to each peak of the chromatographic profile obtained (Figure 7A). The significantly active fractions (between 17 and 29 min) were identified and reported. These active fractions were assayed in the presence (black bars) or in the absence (white bars) of NMDA (Figure 7B) and, then, were successively analyzed using a High Performance Liquid Chromatography-tandem Mass Spectrometry (HPLC-MS/MS) technique. In detail, from the interpretation of the fragmentation spectra, we observed that some of the molecules have a molecular scaffold comparable to that of other molecules found in olive pomace extract. In particular, from the fragmentation pattern analysis of signals at *m*/*z* ratios of 573, 529, and 747 (indicated with the Y arrow in Figure 7A) the same fragment ions 483, 441, and 423 were found, characteristic of proanthocyanidins, which is consistent with dimers or trimers of catechin/epicatechin and/or gallocatechin/epigallocatechin [41]. Another signal at an *m*/*z* ratio of 407 (indicated with the X arrow in Figure 7A) has a fragmentation pattern containing ions 389, 375, 357, 313, and 151 attributable to a hydroxy derivative of loganin [42].

**Figure 8:**
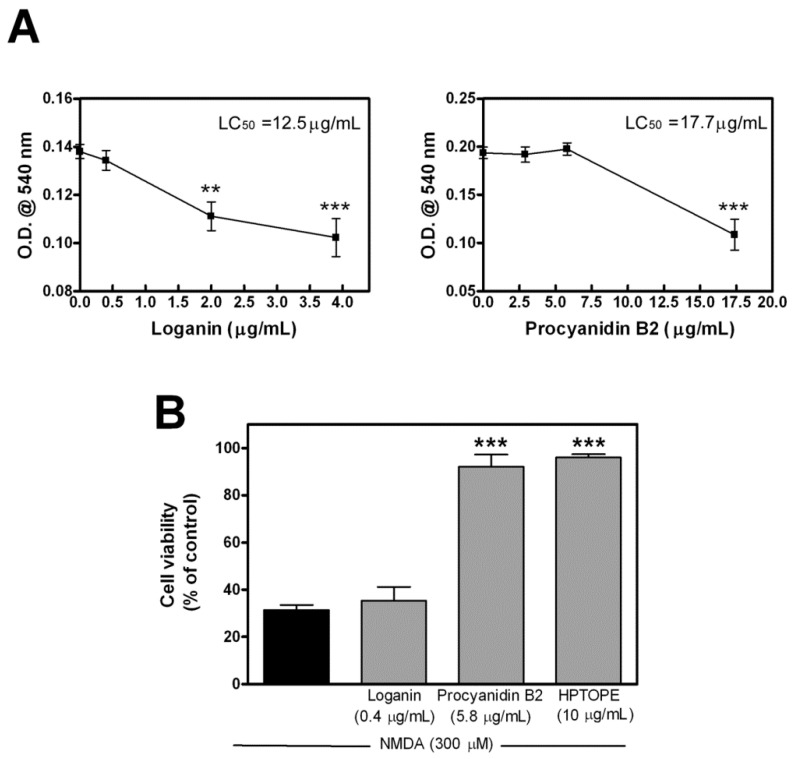
Cell viability of cortical neurons following NMDAR stimulation in the presence of loganin or procyanidin B2. After 14 DIV, neurons were exposed for 24 h to the indicated stimuli, and cell viability was evaluated by NRU assay. (**A**) Cell viability was evaluated following neuron exposure to increasing concentrations of both loganin and procyanidin B2. Data are means ± SEM from two experiments in triplicate. ** *p* < 0.01 and *** *p* < 0.001, according to ANOVA, followed by Tukey’s post-hoc test. LC_50_ was calculated using the online free tool “Quest Graph™ LC_50_ Calculator”, AAT Bioquest, Inc., 9 Sep. 2020, https://www.aatbio.com/tools/lc50-calculator. (**B**) Cell viability was evaluated following neuron exposure to NMDA in the absence or in the presence of the indicated stimuli. Data are means ± SEM from three experiments in triplicate. *** *p* < 0.001, according to ANOVA, followed by Tukey’s post-hoc test.

Therefore, we tested the viability of neurons in NMDAR overstimulation conditions in the presence or absence of the maximum noncytotoxic concentrations (Figure 8A) of loganin or procyanidin B2. The results shown in Figure 8B indicate that, while loganin failed to protect neurons, procyanidin B2, just like HPTOPE, was able to protect cells from calcium overload-induced cell death. This result suggests that procyanidin B2 could be the bioactive molecule responsible for the effect of HPTOPE.

## 3. Discussion

In this study we found that bioactive molecules, extracted from olive pomace by means of a very reproducible high-pressure and temperature extraction method, prevent the death of murine cortical neurons induced by intracellular Ca^2+^ overloading triggered by the overstimulation of NMDAR. Particularly, detailed investigations of the effect of HPTOPE treatment show that this extract selectively reduces the cell Ca^2+^ influx mediated by NMDAR activation on neurons exposed to cytotoxic concentrations of the glutamate analog NMDA, resulting in a significant protection of neuronal viability. Moreover, we demonstrated that the regulation of the calcium influx exerted by HPTOPE results in the significant prevention of overactivation of the calcium-dependent protease calpain. Since the uncontrolled activation of calpain is lethal to neurons, as well as to other cell types [22,43], we can hypothesize that the preservation of neuronal viability by cell treatment with HPTOPE could be related to a preservation of physiological calpain activity. In this context, it is possible to find a relationship between calpain activity and neuronal cell death by evaluating the occurrence of a specific degradation pattern of nNOS [39,40,44]. Particularly, when we treated neurons with cytotoxic concentrations of NMDA in the presence of HPTOPE, we observed a trend for an accumulation of the active 130-kD form produced by calpain. This limited proteolytic processing and activation of nNOS has been previously proposed as a neuroprotective mechanism that prevents the overproduction of NO [39,40]. Hence, we hypothesize that one of the molecular events involved in the preservation of neuronal viability induced by HPTOPE consists of the reduction of Ca^2+^ influx regulating the extent of calpain activation.

Since our mass spectrometry analyses identified that bioactive molecules belonging to the class of proanthocyanidins, specifically dimers/trimers of catechin/epicatechin and/or gallocatechin/epigallocatechin, are present in HPTOPE, and since it is known that these molecules can play neuroprotective effects against glutamate-induced excitotoxicity [9,45,46], we tested the effect of procyanidin B2 on neuronal cell viability in conditions of NMDAR overactivation. Our results indicate that this molecule promotes a protective effect on neurons similar to that exerted by HPTOPE. Hence, we hypothesized that proanthocyanidin-derived molecules could be among the main molecules responsible for the activity of HPTOPE. In support of this hypothesis, several studies have shown that the release of proanthocyanidins from the vegetal matrix is related to an increase in extraction temperature [47]. In general, indeed, an increase in temperature and pressure, during the extraction process, leads to a decrease in the viscosity and surface tension of solvents, promoting an increase of compound mass transfer rates from the matrix [48]. Thus, the changes in extract composition could be due to different compounds coextracted during the process under different operating conditions [49]. An increase in temperature can lead to a high solubility of some molecules and, on the other hand, can lead to less solubility of others, due to changes of their structure [50]. Of relevance, in a more recent study, Vergara-Salinas et al. [51] reported that the recovery of proanthocyanidins augments with temperature, especially at temperatures higher than 100 °C, decreasing the number of free anthocyanins (monomeric pigments). The authors observed that the temperature not only changes the proanthocyanidin content but, also, that a high content of procyanidin B2 in their extract prepared at 150 °C was detected. All these considerations highlight the importance of the selected extraction conditions used for obtaining the bioactive properties of HPTOPE that we observed to be absent in SLE obtained at room temperature. In this respect, it is also crucial to emphasize that, in HPTOPE, despite the high extraction temperature (180 °C), no traces of 5-(hydroxymethyl)furfural were detected by HPLC-MS/MS analysis (Figure S1). The absence of this furanic compound, formed during the Maillard reaction, could indicate that no degradation reactions occurred during the extraction process [52]. This could be due to the presence of high ethanol concentrations (50% *v*/*v*) and to the absence of oxygen in the reaction vessel.

However, further investigations are ongoing to obtain more information from the structural analysis of the molecules present in HPTOPE and, also, to find out the possible additive effects among them. Indeed, our results suggest that, probably, the concentration of procyanidin B2 in HPTOPE is lower than that used in our experimental conditions.

In conclusion, our results provide evidence that HPTOPE is endowed with the ability to maintain intracellular calcium homeostasis, protecting neurons from adverse effects induced by NMDAR overactivation, including aberrant calpain activation. Considering the widespread and devastating effects that derive from the alteration in [Ca^2+^]_i_ and the pathological activation of calpain, both considered among the most important neurodegenerative factors [53,54], currently, the development of effective and reliable approaches to prevent these events represents a crucial target to counteract neurodegenerative diseases.

Given the important biochemical and functional properties of HPTOPE on primary neuronal cultures, the possibility of extracting it easily and, as in our experimental conditions, without producing toxic compounds for neurons, it is possible to hypothesize its eventual use for new prophylactic applications and therapeutic interventions for neurodegeneration.

Our future goals are to identify in deep any biologically active components of HPTOPE. Particularly, a quantitative analysis of the main phytoconstituents, especially focusing on proanthocyanidins, will be performed. Thus, we will try to characterize each biologically active element in terms of concentration, interaction with other elements, possible adverse side effects, and the capability to cross the blood–brain barrier. In our opinion, this characterization will be fundamental for identifying new and more effective therapeutic approaches towards neurodegenerative diseases.

## 4. Materials and Methods

### 4.1. Reagents and Antibodies

NMDA, AMPA, glutamate, MK-801 hydrogen maleate, and poly-L-lysine were purchased from Sigma-Aldrich (St. Louis, MO, USA). ECL Select™ Western Blotting Detection Reagent and Amersham™ Protran^®^ Premium 0.45-µm nitrocellulose were obtained from GE Healthcare (Chicago, IL, USA). Calcium Green™-1AM, *t*-butyloxycarbonyl (*t*-BOC)-Leu-Met-7-amino-4-chloromethylcoumarin (CMAC), Neurobasal™ medium, B-27 supplement, Glutamax^®^, penicillin, and streptomycin solution were purchased from Life Technologies Italia (Milano, Italy). Mouse monoclonal anti-nNOS antibody was purchased from BD Transduction Laboratories (San Jose, CA, USA). Mouse monoclonal anti-β-actin antibody was from Santa Cruz Biotechnology (Heidelberg, Germany). Horseradish peroxidase (HRP)-linked anti-mouse and anti-rabbit secondary antibodies and protease and phosphatase inhibitor cocktails were purchased from Cell Signaling (Danvers, MA, USA).

### 4.2. Cell Culture

Primary neuron cultures were prepared from cerebral cortices of E18 0-day-old mouse embryos recovered from CO_2_-anaesthetized pregnant C57BL/6J, as reported in [55]. Briefly, embryos were removed, micro-dissected, and brain cortex pieces were dissociated by enzymatic digestion in trypsin 0.25% for 20 min at 37 °C and then triturated with a fire-polished Pasteur pipette. Dissociated neurons were plated onto 0.1-mg/mL poly-L-lysine-coated cell culture supports. Cells were maintained at 37 °C in a 5% CO_2_ humidified atmosphere in a culture medium consisting of Neurobasal™ medium, supplemented with B-27 (1:50 *v*/*v*), Glutamax^®^ (1% *v*/*v*), 10-U/mL penicillin, and 100-µg/mL streptomycin. Fifty percent of the medium was changed weekly. No antimitotic medium agent was used to control glial proliferation, because the application of serum-free medium limits the growth of non-neuronal cells. Neurons were allowed to grow functional and structural mature networks before use after 14 DIV.

### 4.3. Extraction of Bioactive Molecules from Olive Pomace of Taggiasca Cultivar

Bioactive molecules were extracted from the olive pomace of Taggiasca cultivar by means of the HPT reactor (model 4560, PARR Instrument Company, Moline, IL, USA). HPTOPE was selected because of the good result obtained in polyphenols recovery if compared with other extraction methodologies, such as conventional solid–liquid extraction, microwave-assisted extraction, and ultrasound-assisted extraction [7,56]. Extraction parameters were selected based on previous works [7,25]. During the extraction, pressure and temperature inside the reactor reached 25 bar and 180 °C, respectively. After extraction, the liquid phase rich in polyphenols was separated from the exhausted olive pomace by centrifugation at 6000× *g* for 10 min. Then, 10 mL of the olive pomace extract was dried by a rotary evaporator (Laborota, Heidolph, Germany), and the dried sample was recovered using 1 mL of ethanol/water 1:1 (*v/v*). This extract, stored at −20 °C, resulted stably in terms of total polyphenols concentration and biological activity up to 6 months.

A classic SLE was obtained after the extraction of olive pomace at room temperature for 24 h using the same solid–liquid ratio and solvent composition used for HPTOPE. The HPTOPE and SLE extracts were stored at −20 °C until further analysis. On HPTOPE and SLE, the total polyphenol concentration was determined using the Folin-Ciocalteu assay and was expressed as mg of caffeic acid equivalents (CAE) per mL of solvent (mg_CAF_/mL). Single phenolic compounds were quantified using a HPLC Agilent 1100 Series (Palo Alto, CA, USA), equipped with a C18 reverse-phase column (Model 201TP54, Vydac, Hesperia, CA, USA) and coupled with a diode array detector and using the corresponding phenol standard solutions following the methodology described in [25]. Different preparations of HPTOPE were performed but with the same method of extraction [25,57]. The concentrations in total polyphenols and single phenolic compounds in HPTOPE after the extraction at high pressure and high temperature were total polyphenols: 19.2 mg_CAE_/mL, tyrosol: 0.51 mg/mL, caffeic acid: 0.23 mg/mL, coumaric acid: 0.18 mg/mL, oleuropein: 3.59 mg/mL, and apigenin: 0.08 mg/mL.

### 4.4. Cell Viability Assay

Cortical neurons (2.5 × 10^4^, seeded in a 96-well microplate) were exposed to the stimuli specified elsewhere, and after 24 h at 37 °C in a 5% CO_2_ humidified atmosphere, cell viability was measured using the NRU assay, as reported by Repetto et al. [58].

### 4.5. Immunoblot Analysis

Cortical neurons (3 × 10^5^), seeded in 35-mm culture dishes and treated as described elsewhere, were lysed by sonication in 150 μL of 50-mM Tris/HCl, pH 7.4, containing 150-mM NaCl, 1-mM EDTA, 1% Triton X-100, and protease and phosphatase inhibitor cocktails diluted 1:100. The lysates were centrifuged at 10,000× *g* for 10 min at 4 °C, the supernatants were collected, and protein quantification was assayed by the method of Lowry. Samples were then heated for 5 min at 95 °C, and aliquots of each sample (20 µg/lane) were separated by SDS/PAGE (8%), followed by immunoblotting. Nitrocellulose membranes were successively incubated for 16 h at 4 °C with primary antibodies: anti-nNOS (1:2500) and anti-β-actin (1:1000). Peroxidase-conjugated secondary antibody (1 h at 22 °C) was anti-mouse (1:5000). Immunoreactive signals were developed using ECL Select^TM^ Western Blotting Detection Reagent, acquired and quantified using ChemiDoc™ XRS equipped with Quantity One Image Software 4.6.1 (Bio-Rad Laboratories Srl, Segrate (MI), Italy).

### 4.6. [Ca^2+^]_i_ Assay

Cortical neurons (2.5 × 10^4^, seeded in a 96-well microplate) were incubated in HEPES buffer (NaCl 128 mM, KCl 2.4 mM, MgSO_4_ 1.2 mM, CaCl_2_ 1.2 mM, KH_2_PO_4_ 1.2 mM, glucose 10 mM, and HEPES 10 mM, pH 7.3–7.4) containing 10-μM Calcium Green™-1AM. After 40 min at 37 °C, cells were washed twice with HEPES buffer and then exposed to the stimuli specified elsewhere in the absence or in the presence of either HPTOPE extract or MK-801. The fluorescence intensity (excitation 485 nm and emission 535 nm) was measured every 10 s for 4 min using the top reading mode in the fluorescence multilabel reader LB 940 Mithras (Berthold Technologies, Baden Württemberg, Germany). Variations of the fluorescence values were calculated as the difference between each fluorescence value recorded and the one measured at time zero. The values obtained were then subtracted from the relevant control values.

### 4.7. Assay of Intracellular Calpain Activity

Cortical neurons (2.5 × 10^4^_,_ seeded in a 96-well microplate) were incubated at 37 °C for 20 min with 50-μM *t*-BOC-Leu-Met-CMAC fluorogenic calpain substrate in HEPES buffer. Cells were then washed twice with HEPES buffer to remove substrate excess, exposed to NMDA in the absence or in the presence of HPTOPE extract, and the fluorescence emission was detected at time zero and after 1 h with a Mithras LB 940 plate reader. The excitation/emission wavelengths were 355/485 nm, respectively.

### 4.8. HPLC Separation and HPLC-MS/MS Analysis

Firstly, HPTOPE was submitted to a pretreatment: 10 min at 37 °C, 2 min of vigorous agitation on vortex, and finally, 10 min at 6000× *g* to separate the insoluble fraction of the extract. Secondly, 20 µL of the supernatant was put together with 30 µL of water, and the resulting 50 µL were manually injected into an analytical HPLC Agilent 1260 equipped with a Luna^®^ C18 column (pore size 300 Å, particle size 5 μm, and 3.9 mm i.d. × 150 mm). The chromatographic method consisted of the following 60-min gradient: 0–5 min 0% B, 5–10 min 5% B, 10–30 min 30% B, 30–40 min 40% B, 40–45 min 48% B, 45–55 min 70% B, and 55–60 min 100% B at a 1-mL/min flow rate, where A is H_2_O containing 1% acetic acid and B is CH_3_CN/CH_3_OH (1:1). The detector was set at 220/254 nm. Fractions were collected every 60 s, dried with a centrifugal vacuum concentrator, and reconstituted with 50-mM sodium borate, pH 7.5 (100 µL).

After the cell viability assay on cortical neurons, only the bioactive fractions were submitted to HPLC-MS/MS analysis. The chromatographic separation was carried out by means of the Agilent 1100 µHPLC equipped with a micro-autosampler and a Zorbax C_18_ column (pore size 300 Å, particle size 3.5 µm, and 0.5 mm i.d. × 150 mm) kept at 30 °C. Injection volume was 8 µL. The chromatographic method consisted of the following 60-min gradient: 0–5 min 10% B, 5–50 min 100% B, and 50–60 min 100% B at a 20-µL/min flow rate, where A is H_2_O containing 0.1% FOA (formic acid) and B is CH_3_OH containing 0.1% FOA. The detector was set at 220/280 nm. Finally, the HPLC was coupled with the mass spectrometer (HPLC-ESI-MS/MS) to value qualitatively the compound of interest.

The instrument utilized was a mass spectrometer with an orthogonal electrospray ion source (ESI) and high capacity ion trap (Agilent 1100 MSD XCT ion trap). All the parameters were established in order to obtain the best ionization of the fraction’s components. Analysis was performed in ion-charged mode control with a selected target at 100,000 and an accumulation time of 300 ms. The operative parameters were capillary voltage: 3.8 V, nebulizer pressure: 20 psi, drying gas: 8 L/min, dry temperature: 360 °C, rolling averages: 3, and fragmentation amplitude: 0.8 V.

All the mass spectra were acquired in full-scan and in MS-MS modality, breaking between the most abundant species under every peak. The acquisition was performed on negative and positive ions in the mass range of 50–1200 and analyzed using integrated Agilent Data Analysis software (LC/MSD Trap Software version 5.3).

### 4.9. Statistical Analysis

Data were presented as mean ± SEM. Significance of the difference was analyzed by ANOVA, followed by post hoc Tukey’s test or, where indicated, by *t*-test, using the Prism 4.02 software package (GraphPad Software, San Diego, CA, USA), with statistical significance taken at *p* < 0.05.

## Supplementary Materials

The following are available online, Figure S1: The HPLC-MS/MS analysis of 5-(hydroxymethyl)furfural (HMF) standard was performed in the positive ion mode. The panel A shows the extracted ion current (XIC) of the corresponding molecular ion at *m*/*z* 127. Extracting the same *m*/*z* ratio from the analysis of the HPTE extract (panel B) it was not possible to obtain any signal. For the standard molecules, the mass spectrum at RT 12.6 min is reported (panel C). In the panel D the mass spectrum at the same RT is shown for the HPTE extract; it is evident the absence of *m*/*z* 127 ion.
