# Supplementary material for: A Bioactive Olive Pomace Extract Prevents the Death of Murine Cortical Neurons Triggered by NMDAR Over-Activation
Source: Molecules. 2020 Sep 24;25(19):4385. doi: 10.3390/molecules25194385 (PMC7839963; doi:10.3390/molecules25194385)
Supplement: Supplementary file 1 [file molecules-25-04385-s001.pdf]

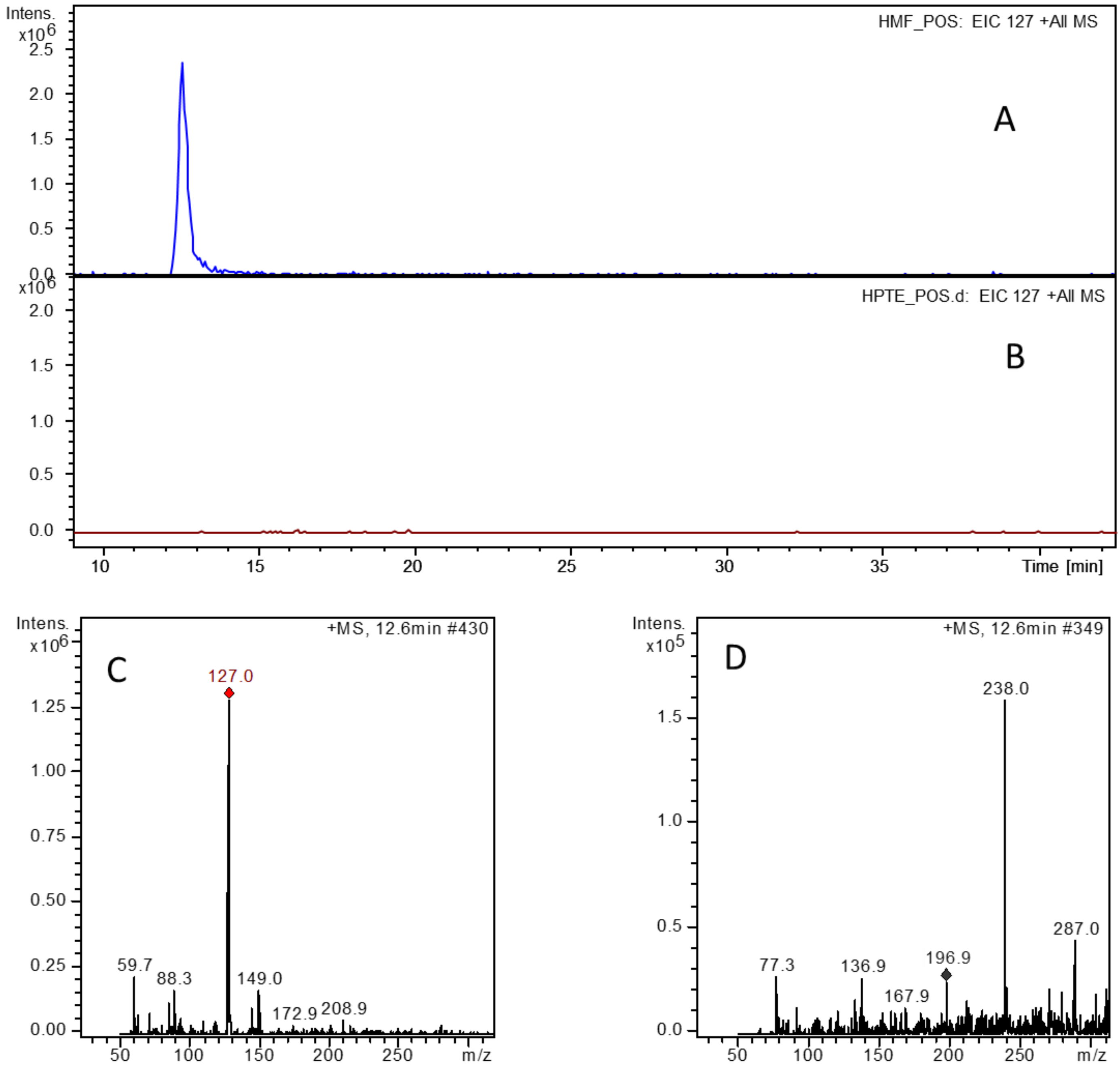

Fig. S1. The HPLC-MS analysis of 5-(hydroxymethyl)furfural (HMF) standard was performed in the positive ion mode. The panel A shows the extracted ion current (XIC) of the corresponding molecular ion at m/z 127. Extracting the same m/z ratio from the analysis of the HPTE extract (panel B) it was not possible to obtain any signal. For the standard molecules, the mass spectrum at RT 12.6 minutes is reported (panel C). In the panel D the mass spectrum at the same RT is shown for the HPTE extract; it is evident the absence of m/z 127 ion.
